# Measurement of Cancer Cell Growth Heterogeneity through Lentiviral Barcoding Identifies Clonal Dominance as a Characteristic of *In Vivo* Tumor Engraftment

**DOI:** 10.1371/journal.pone.0067316

**Published:** 2013-06-26

**Authors:** Olivier Nolan-Stevaux, Donato Tedesco, Seamus Ragan, Mikhail Makhanov, Alex Chenchik, Astrid Ruefli-Brasse, Kim Quon, Paul D. Kassner

**Affiliations:** 1 Oncology Research, Amgen, South San Francisco, California, United States of America; 2 Cellecta Inc., Mountain View, California, United States of America; 3 Genome Analysis Unit, Amgen, South San Francisco, California, United States of America; 4 Therapeutic Innovation Unit, Amgen, Seattle, Washington, United States of America; The University of Texas M.D. Anderson Cancer Center, United States of America

## Abstract

Advances in the fields of cancer initiating cells and high-throughput *in vivo* shRNA screens have highlighted a need to observe the growth of tumor cells in cancer models at the clonal level. While *in vivo* cancer cell growth heterogeneity in xenografts has been described, it has yet to be measured. Here, we tested an approach to quantify the clonal growth heterogeneity of cancer cells in subcutaneous xenograft mouse models. Using a high-throughput sequencing method, we followed the fate *in vitro* and *in viv*o of ten thousand HCT-116 cells individually tagged with a unique barcode delivered by lentiviral transduction. While growth *in vitro* was less homogeneous than anticipated, we still find that 95% of the final cells derived from 80% of the original cells. In xenografts, however, 95% of the retrieved barcoded cells originated from only 6% of the initially injected cells, an effect we term “clonal dominance”. We observed this clonal dominance in two additional xenograft models (MDA-MB-468 and A2780^cis^) and in two different host strains (NSG and Nude). By precisely and reproducibly quantifying clonal cancer cell growth *in vivo*, we find that a small subset of clones accounts for the vast majority of the descendant cells, even with HCT-116, a cell line reported to lack a tumor-initiating compartment. The stochastic *in vivo* selection process we describe has important implications for the fields of *in vivo* shRNA screening and tumor initiating cells.

## Introduction

In recent years, xenograft mouse models of cancer have been used to probe fundamental questions in tumor biology as diverse as the existence of cancer initiating cells or the feasibility of identifying novel cancer target genes using *in vivo* shRNA drop-out screening approaches. In both fields, however, the relatively poor understanding of the growth dynamic of xenograft models caused confusion.

First, results from serial dilution experiments, in which very low numbers of cancer cells are injected subcutaneously into mice have been used to support [1], [2] or refute [3] the existence of rare cancer initiating cells inside heterogeneous pools of cancer cells in solid tumors [4]. However, cancer cells in tumors do not exist by themselves but are surrounded by other cancer cells. Thus, if a few cancer cells are injected in a mouse and fail to grow, it may reflect their lack of cancer initiating potential; or more prosaically, the fact that they were not in an optimal environment, surrounded by other cancer cells (tumor initiating or not). Tracking the behavior of the putative cancer initiating cells surrounded by putative non-cancer initiating cells would provide much needed clarity.

Second, methodologies using pooled libraries of lentiviral vectors encoding hundreds of shRNA triggers have been pioneered to identify potential novel cancer-promoting genes *in vivo* [5], [6]. However, while *in vitro* pooled shRNA drop-out screens (for a recent example, see [7]), and *in vivo* pooled shRNA enrichment screens aimed at identifying tumor-suppressors or growth inhibitory mechanisms have been successful [8], [9], *in vivo* shRNA drop-out screens have not been widely replicated. Here too, a better understanding of the growth heterogeneity in xenograft models would help interpret and predict results from such screening approaches. Remarkably, while spatial phenotypic heterogeneity has been documented in xenograft cancer models [10], [11], clonal cancer cell growth heterogeneity in xenograft models has never been measured.

Here, we have used a method of lentiviral barcode tagging to accurately and simultaneously measure the growth characteristics of thousands of individual cancer cells inside a pool of untagged cancer cells grown *in vitro* or injected subcutaneously *in vivo* in severely immuno-deficient mice. Our results demonstrate the remarkable heterogeneous growth of cancer cells in several xenograft models, whereby small numbers of individual cancer cell clones take over an initially evenly-distributed and heterogeneous cell population, an effect we have termed “clonal dominance”. As a result of our observations, we propose a new clonal cell tracking method to circumvent the confounding effect of clonal dominance in the context of pooled *in vivo* shRNA drop-out screens. We also recommend use of this method to measure the contribution of putative cancer initiating cells sub-populations, not in isolation, but within a heterogeneous cancer cell population.

## Materials and Methods

### Ethics Statement and Mouse Strain

Mice were cared for in accordance to the *Guide for the Care and Use of Laboratory Animals, 8^th^* Edition from the National Institute of Health. Animals were housed at a facility internationally accredited by the Association for Assessment and Accreditation of Laboratory Animal Care (AAALAC), in ventilated micro-isolator housing. Animals had *ad libitum* access to feed and water via automatic watering system. Animals were maintained on a 12 hr:12 hr light:dark cycle, in rooms at 22°C and 45% humidity. Our research protocol and animal housing plan were approved by the Amgen South San Francisco Institutional Animal Care and Use Committee (Amgen South San Francisco IACUC, Protocol #2011-01108). Eight week-old female NOD/SCID IL2rg mice (NSG) (Jackson Laboratories strain #5557) and ten week-old female Athymic Nude mice (Charles River strain #490) were used in this study.

### Lentiviral Library Titer Determination by FACS

The titer of the pooled lentiviral library was determined directly in HCT-116 cells by FACS measurement of the percentage of mCherry positive cells from a serial dilution of the lentiviral pool. Briefly, titrations of the lentiviral pool were added to 1.5×10^5^ HCT-116 cells in growth media (McCoy’s 5A, 10% FBS) containing DEAE Dextran (MP Biomedicals, Catalog # 195133) at 10 µg/mL. Cells were exposed to virus for 16 hours and infection media was aspirated and replaced with complete growth media without DEAE Dextran. Cells remained in culture for another 48–72 hours and were trypsinized, washed with Dulbeco’s PBS, and fixed in a 2% paraformaldehyde solution. Fixed cells were analyzed for the percentage of mCherry positive cells by FACS (Becton Dickinson LSRII). The multiplicity of infection (MOI) and titer, reported as transducing units per ml (TU/mL), were determined from the percentage of transduced cells and the volume of lentiviral stock used. The MOI was first determined using the equation % Transduced cells = 100*(1– e^-(MOI)^) and the titer value was determined using the equation Titer = [MOI × # cells at infection]/vol (ml of virus) = TU/ml [12]–[15]. For this viral pool, 10 µL of virus resulted in 12.9% mCherry positive cells and the calculated titer of 2×10^6^ TU/mL was used to determine the appropriate volume of virus for subsequent experiments at selected MOI’s.

### Calculations of Transduction Efficiency and Number of Lentiviral Inserts Per Cell

Virus particles are expected to distribute randomly into individual cells and the percentage of infected cells at a given MOI (where MOI = Transducing Units/Cell) can be estimated by a Poisson distribution where the percent of infected cells is equal to 100*(1– e^-(MOI)^) (Table 1). The probability of any number of virus particles in a given cell is, thus, given by the Poisson equation p(v) = (M^v^e^-v^)/v!, where M = MOI and v is the number of virons infecting the cell. These formulas can be used to calculate the titer of a viral pool in a given cell line and estimate the percentage of cells infected with any number of virons (Table 2).

**Table 1:** Percentage of transduced cells at a given MOI.

MOI	0.02	0.05	0.1	0.2	0.3	0.5	0.7	1
Transduced Cells (%)	2	4.9	9.5	18.1	25.9	39.4	50.3	63.2

**Table 2:** Percentage of cells carrying n lentiviral insertions for a given MOI.

MOI	Transduction Efficiency (%)	% of the infected cells with n lentivirus		
		n = 1	n = 2	n = 3
0.1	9.52	95.06	4.73	0.21
0.2	18.12	90.29	9.04	0.61
0.3	25.92	85.73	12.85	1.27
0.5	39.35	77.08	19.26	3.2
0.7	50.34	69.05	24.18	5.64
1	63.21	58.2	29.09	9.7

### Lentiviral Infection Procedure for KE-U6-TET Library in HCT-116

HCT-116 cells (Colon Cancer Cell Line - ATCC #CCL-247) with a doubling time of 21 Hrs. were cultured in complete growth medium [McCoy’s 5A medium (Life Technologies #16600-082), 10% Tet System Approved FBS (Clontech #631101), 0.1 mg/ml Normocin (Invivogen #ant-nr-1)]. KE-U6-TET, a lentiviral library containing 27,500 individual Tet-inducible barcoded shRNA sequences with a titer of 2×10^6^ Transduction Units (TU)/ml was used in conjunction with 10 µg/ml DEAE Dextran (MP Biomedicals #195133) to achieve an (MOI) of 0.1, for which the probability of infecting a cell with more than one lentiviral particle is calculated to be less than 5% (Table 2) [15]. Briefly, 3×10^6^ HCT-116 cells were re-suspended in 8.5 ml of complete growth media supplemented with DEAE Dextran (10 µg/ml) and transduced with 150 µl of KE-U6-TET library, and incubated for 16 hours, resulting in a cell population of 3×10^6^ cells containing ∼ 3×10^5^ cells infected with a single lentivirus and carrying an individual barcode.

### Lentiviral Infection Procedure for Luciferase library in HCT-116, MDA-MB-468 and A2870^cis^

A lentiviral library containing 27 neutral Renilla *Luciferase* shRNAs sequences associated with 2% of the barcodes present in the library was used at an MOI of 0.18, 0.17 and 0.15, respectively in HCT-116, MDA-MB-468 (Breast Cancer Cell Line - ATCC #HTB-132) and A2780*^cis^* (Cisplatin Resistant Ovarian Cancer Cell Line - Sigma-Aldrich #93112517) cells. The projected percentage of infected cells with more than one lentiviral particle is calculated to be less than 10% at these MOIs (Table 2). Briefly, 3×10^6^ cells were re-suspended in 8.5 ml of growth media supplemented with polybrene (5 µg/ml), transduced with the lentiviral library, and incubated for 16 hours, resulting in a cell population of 3×10^6^ cells containing ∼ 5×10^5^ transduced cells, ∼ 90% of which are predicted to be infected with a single lentivirus and carrying a single barcode (Table 2).

### *In vitro* Experiment

16 hours post-transduction with the KE-U6-TET library, samples of 10^5^ HCT-116 cells containing ∼10^4^ individually tagged cells were seeded in triplicate in T175 cell culture flasks and grown continuously for 8 days in complete growth medium, with media replenishment every 2–3 days. For each *in vitro* replicate, all the cells from each T175 flask were used upon harvesting at day 8 for genomic DNA isolation.

### NSG Xenograft Experiment

16 h post-transduction with the KE-U6-TET library, samples of 10^5^ HCT-116 cells containing ∼10^4^ individually tagged cells were admixed with 3×10^6^ non-transduced HCT-116 cells and resuspended in 200 µl of 1∶1 PBS:Matrigel solution (BD Bioscience #356235) prior to subcutaneous injection in 8-week old female NSG mice (stock # 005557, Jackson laboratories). Each xenograft was allowed to grow for 12 days until the resulting subcutaneous tumor reached a size of ∼500 mm^3^. At this point, tumors were harvested for genomic DNA isolation.

### Nude Xenograft Experiments

16 h post-transduction with the Luciferase library, 3×10^6^ cells (HCT-116, MDA-MB-468 or A2780^cis^) were resuspended in 200 µl of 1∶1 PBS:Matrigel solution (BD Bioscience #356235) prior to subcutaneous injection in 10-week old female Nude mice (stock # 490, Charles River Laboratories). HCT-116 and A2780^cis^ xenograft were allowed to grow for 14 days and MDA-MB-468 for 24 days until the resulting subcutaneous tumor reached a size of ∼500 mm^3^. At this point, tumors were harvested for genomic DNA isolation.

### Tet Repression of shRNA Expression in the Absence of Doxycycline and Induction with Doxycycline

The effectiveness of the TET repressor element in the vector was assessed over a 15 day experiment. Three independent infections of 9×10^7^ HCT116 cells in growth media (McCoy’s 5A, 10% FBS) containing DEAE Dextran (MP Biomedicals, catalog # 195133) at 10 µg/mL were performed with the 27.5 k shRNA library at an MOI = 0.3 in Corning Cell Stack 10 vessels (catalog # 3271). To maximize representation of the library, each shRNA was introduced in ∼1000 independent cells. After 24 hours, infection media was aspirated and replaced with complete growth media with 2 µg/mL puromycin (InvivoGen, catalog# ant-pr-5) to begin selection for infected cells. 72 hours after transduction, a cell pellet of ∼ 9×10^7^ cells was collected for reference for each infection triplicate (Day 0 sample). 9×10^7^ cells were re-seeded and maintained in culture with complete media with 2 µg/mL puromycin. Cells were passaged at 3 day intervals out to 15 days, reseeding 9×10^7^ cells and maintaining selective pressure with puromycin for the duration of the experiment. Cell pellets for triplicate samples from day 0 and 15 were submitted for sequencing of the DNA barcodes by high-throughput sequencing. Cells in which shRNA expression was induced were treated after re-seeding 9×10^7^ cells at Day 0 with Doxycycline (Sigma-Aldrich, catalog # D-9891) at 0.5 µg/mL for 15 Days.

### Recovery and Quantification of the Barcodes

Probes were prepared for high throughput sequencing on Illumina HiSeq2000 following Cellecta’s protocol (http://www.cellecta.com/resources/protocols). The set of barcodes used for construction of the library consisted of 27,500 18-nucleotide individual long barcodes, perfectly balanced in AT/GC and purine/pyrimidine, designed using a proprietary Cellecta algorithm. Minimum Hamming distance between barcodes in the set is 4, so up to 3 mutations in an 18-nucleotide sequence can be detected and the corrupted barcodes rejected, providing appropriate level of protection for the current accuracy of Illumina sequencing technology. Barcode ID numbers were encoded in the barcode sequence using quaternary numerical system (A-0, T-1, G-2, C-3), so alignment procedures for barcode deconvolution were not needed. Using Cellecta’s conversion algorithm, barcode ID numbers were extracted from each correct barcode and the abundance of each barcode in the sequenced sample measured. With this procedure, the complexity of calculation is not dependent on the complexity of the library. The complexity is O(n) were n is number of reads in the sequenced probes. FASTQ and qseq files from the Illumina HiSeq2000 machine were used in the analysis.

### Clone Size Estimation

For each sample, the number of barcode counts equivalent to one single transduced cell was calculated based on total number of barcode counts in the sample and total number of transduced cells in the sample (the latter estimated according to genomic DNA recovery and transduction MOI, and confirmed by PCR probe yield). To estimate the size of each clone in the transduced cell population (number of cells carrying the same barcode), barcode counts were then normalized to the calculated single-cell barcode count.

## Results

### Measuring the Clone Recovery Rate *in vivo*

**Figure 1:**
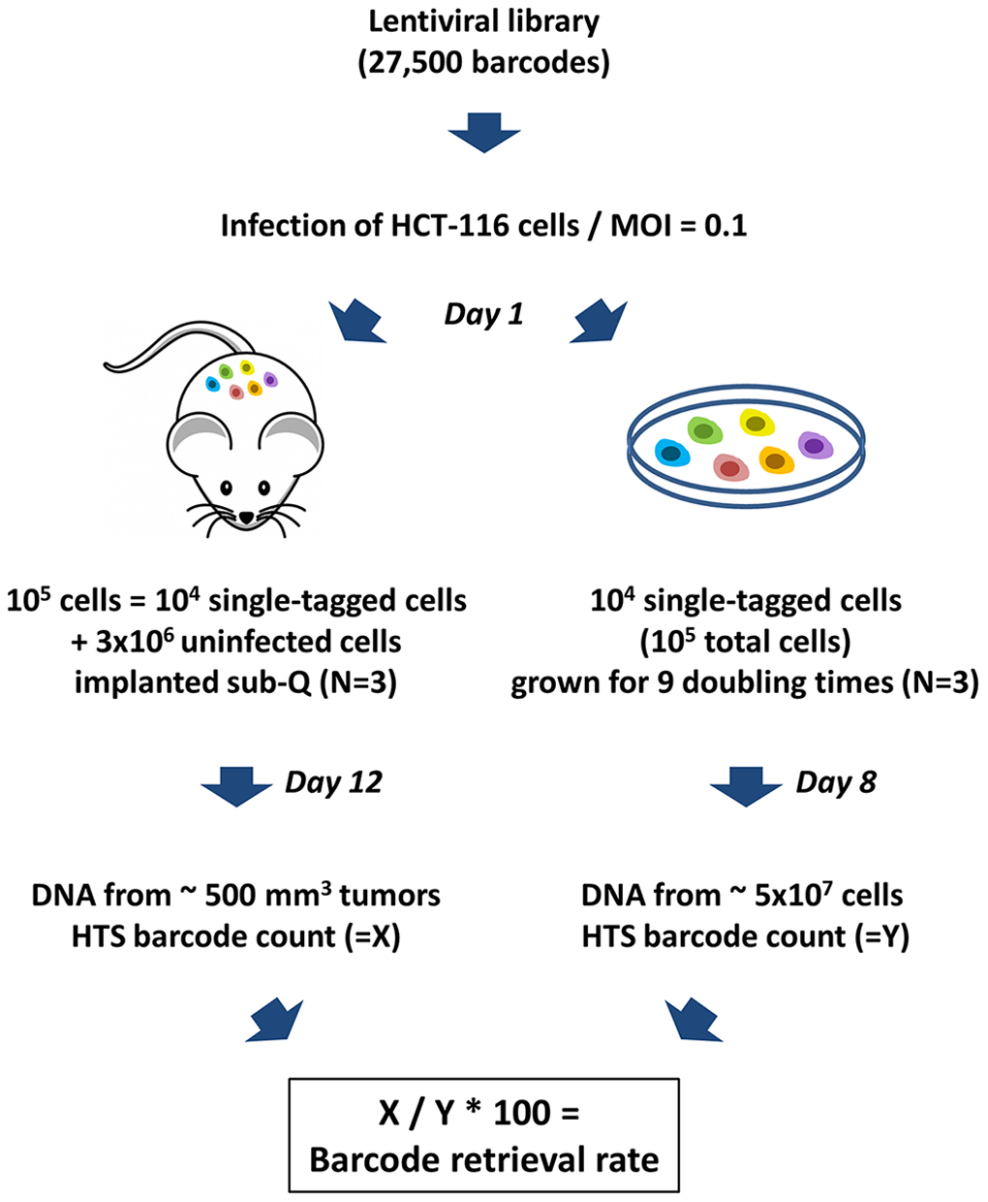
Tracing individually-labeled HCT-116 cancer cell clones *in vitro* and *in vivo.* A lentiviral library containing 27,500 unique barcodes was used to transduce HCT-116 cells at an MOI of 0.1. Pools of 10^5^ cells, corresponding to 10^4^ individually tagged cells were either admixed with 3×10^6^ cells and implanted subcutaneously into NSG mice or grown in culture *in vitro*. Cells were harvested after 9 days and tumor removed after 12 days, and total DNA preparations from cells or tumors were subjected to the barcode retrieval procedure.

In order to track clones originated by individual cells inside a cancer cell population, we infected HCT-116 colorectal cancer cells with a lentiviral library containing 27,500 independent inducible shRNA sequences, each associated with an individual 18 nucleotide barcode. We infected 3×10^6^ HCT-116 cells at a Multiplicity of Infection (MOI) of 0.1. Under these infection conditions, Poisson distribution analysis predict a minimal number of dual infection events (<5% of transduced cells) [15] (Table 2). The transduction yielded ∼3×10^5^ individually barcoded cells (Table 1). Three sets of 10^4^ HCT-116 barcoded cells (10^5^ total cells given an MOI of 0.1) were grown in culture *in vitro* for 8 days or about 9 population doublings. Genomic DNA from the three pools of cells grown *in vitro* was subjected to barcode high-throughput sequencing and the size (cell number) of each detected clone in the transduced population was calculated based on the number of times each bar-code was retrieved (Fig. 1).

In parallel, three sets of 10^4^ barcoded HCT-116 cells (10^5^ total cells given an MOI of 0.1) from the same initial viral transduction event were collected 16 h after transduction and individually admixed with 3×10^6^ uninfected HCT-116 cells and diluted in 50% Matrigel, prior to subcutaneous injection in the flank of three severely immune-deficient female NSG mice. Each xenograft was allowed to grow for 12 days until the resulting subcutaneous tumor reached a size of ∼500 mm^3^. Genomic DNA from the three xenograft tumors was subjected to the same barcode recovery and clone size estimation procedures as the *in vitro* samples (Fig. 1).

The *in vivo* clone retrieval rate was calculated as the ratio between the mean number of barcoded clones identified in the three xenograft tumors and the mean number of barcoded-clones identified in the three cell populations grown *in vitro*, independently of the clone size (Fig. 1). As presented in Table 3, the observed clone retrieval rate was almost 60% and the number of clones identified in the *in vitro* setting in each replicate was very close to the predicted value of 10^4^ clones. Of note, when a cell suspension containing Matrigel is injected subcutaneously, a small amount (∼10%) of the injected liquid seeps out of the injection site, accounting for a fraction of the lost barcodes in the *in vivo* setting.

**Table 3:** Barcode recovery rate from HCT-116 cells in NSG mice and *in vitro*.

	# of unique barcode sequences recovered
*In vitro 1*	10,344
*In vitro 2*	11,504
*In vitro 3*	10,034
***In vitro average (Y)***	***10,627***
*In vivo 1*	5,773
*In vivo 2*	6,734
*In vivo 3*	6,288
***In vivo average (X)***	***6,265***
Recovery rate (X/Y*100)	***58.93%***

### *In vivo* “Clonal Dominance” Inside HCT-116 Xenografts

To assess and compare how the barcoded cells divided *in vitro* and *in vivo*, we analyzed the distribution of their barcodes according to how frequently they were retrieved by HTS. The full data set of clones and barcode counts is available (Table S1).

**Figure 2:**
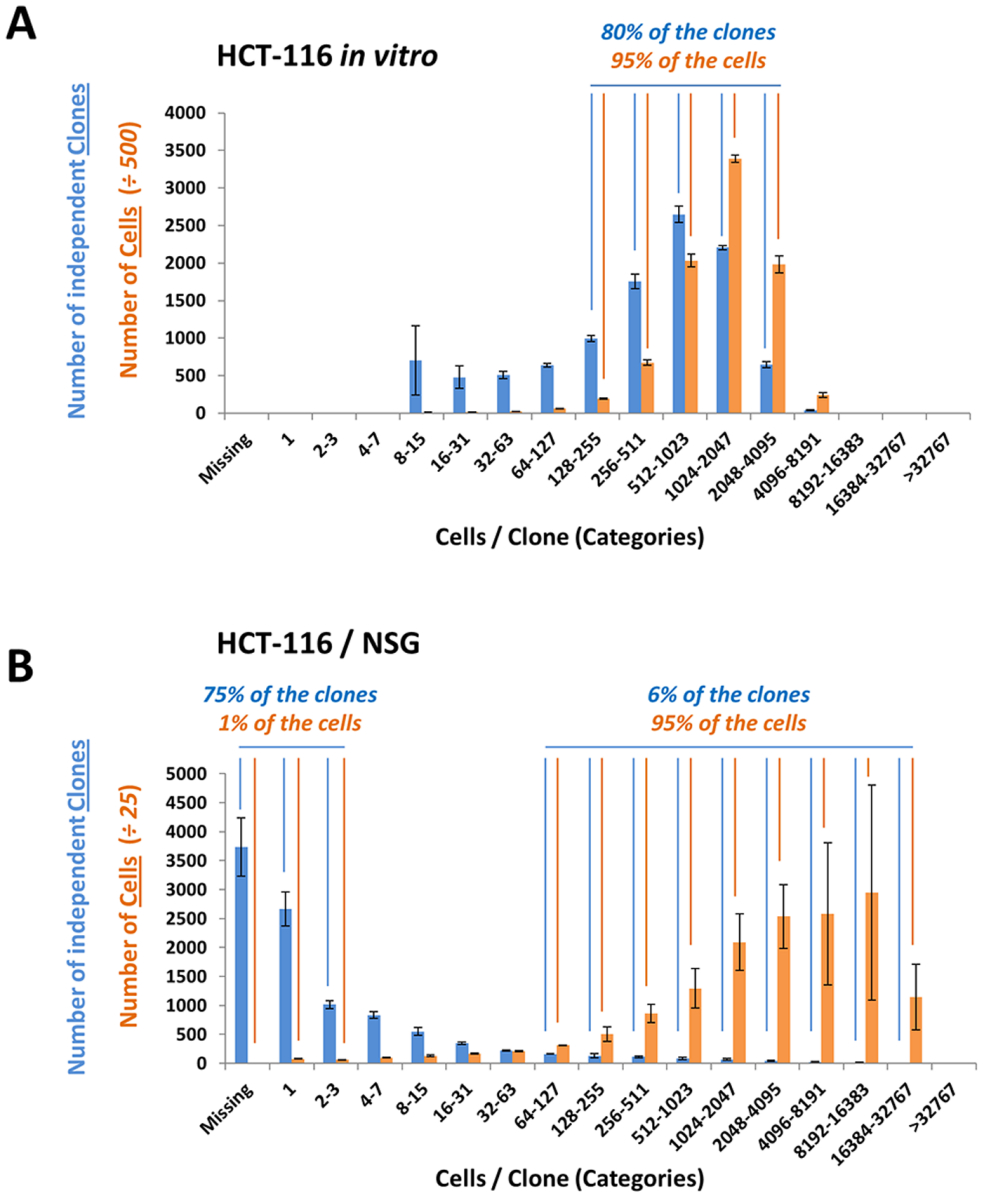
Evidence for clonal dominance: comparison of the distributions of HCT-116 barcoded clones and cells by clone size category *in vitro* and *in vivo* **.** For each graph, the number of independent clones (blue bars) or the total aggregate number of cells (orange bars) is plotted for each clone size category across the X axis. (A) Distribution of HCT-116 barcodes *in vitro*: 80% of the barcodes (clones) were identified in categories “128–255” to “2048–4095”, indicating that 80% of the cells divided between 7 and 12 times; 95% of the cells were identified in categories “128–255” to “2048–4095”, indicating that 95% of the cells belong to clones derived from 80% of the originally transduced cells, which divided between 7 and 12 times. Scale for number of cells was adjusted 500 fold to allow side by side comparisons of clone numbers and cell numbers. (B) Distribution of HCT-116 barcodes *in* *vivo*: 75% of the barcodes are found in categories “missing” to “2–3”, indicating that they either didn’t survive at all, didn’t divide, or divided no more than once and represented only 1% of the retrieved tagged cells. However, only 6% of the bar-codes were located in categories “64–127” to “16384–32767”, indicating that they divided between 6 and 14 times; 95% of the cells accrued in categories “64–127” to “16384–32767”, indicating that 95% of the tagged cells are derived from 6% of the initially barcoded clones that divided the most. Scale for number of cells was adjusted 25 fold to allow side by side comparisons of clone numbers and cell numbers.

In a first chart, we analyzed the clonal distribution of cells grown *in vitro*: we plotted the number of independent clones (Fig. 2A - blue bars) or the total aggregate number of cells (Fig. 2A - orange bars) for each clone size category across the X axis. For example, *in vitro*, there were 2,649 independent clones of a size ranging between 512–1023 cells, indicating that these clones in the ‘512–1023’ cells category must have undergone an average of 8 population doublings (Fig. 2A - blue bar value for the ‘512–1023’ X-axis category). These 2,649 clones contributed a total of 1,017,216 descendant barcoded cells upon *in vitro* culture for 8 days (Fig. 2A - orange bar value for the ‘512–1023’ X-axis category). As proof that *in vitro* clonal growth is mostly homogeneous, 95% of the descendant barcoded cells found after 8 days of *in vitro* culture were derived from 80% of the initially tagged clones and were contained within clone categories ‘128–4095’ indicating at least 7 population divisions.

In a second chart, we analyzed the clonal distribution of cells grown *in vivo*: we again plotted the number of independent clones (Fig. 2B - blue bars) or the total aggregate number of cells (Fig. 2B - orange bars) for each clone size category across the X axis. The *in vivo* distributions look dramatically different from the *in vitro* distributions. *In vivo*, 75% of the clones clustered in the ‘Missing-3′ categories, indicating that 75% of the cells initially injected *in vivo* underwent fewer than two cell divisions (Fig. 2B - blue bars - ‘Missing’ or ‘1’ or ‘2–3′ categories). Confirming that these clones didn’t grow *in vivo*, these clones accounted for only 1% of the total aggregate number of cells within the tagged cell population identified by HTS in the resulting tumor. On the other hand, 6% of the injected cells were able to divide more than five times to generate clones with at least 64 cells (Fig. 2B – blue bars marked 6%). Remarkably, these clones accounted for 95% of the total aggregate number of cells within the tagged cell population (Fig. 2B - orange bars marked 95%), demonstrating very significant growth heterogeneity *in vivo*, where the two most abundantly recovered clones alone (out of an estimated 10,000) underwent 14 population doublings and generated an astounding 7.5% of the total cell tags recovered by HTS in xenografts (Fig. 2B - clones and cells in the ‘16384–32767’ category).

We term this phenomenon “clonal dominance”, whereby a small fraction of the cells implanted *in vivo* divide far more than the vast majority of other clones and contribute overwhelmingly to the descendant cell population. Note that prior studies concluded that the HCT-116 cell line does not contain a stem-cell or tumor-initiating sub-population (see Discussion).

### Excluding an Effect of the Encoded shRNA *in vitro* and *in vivo*

**Figure 3:**
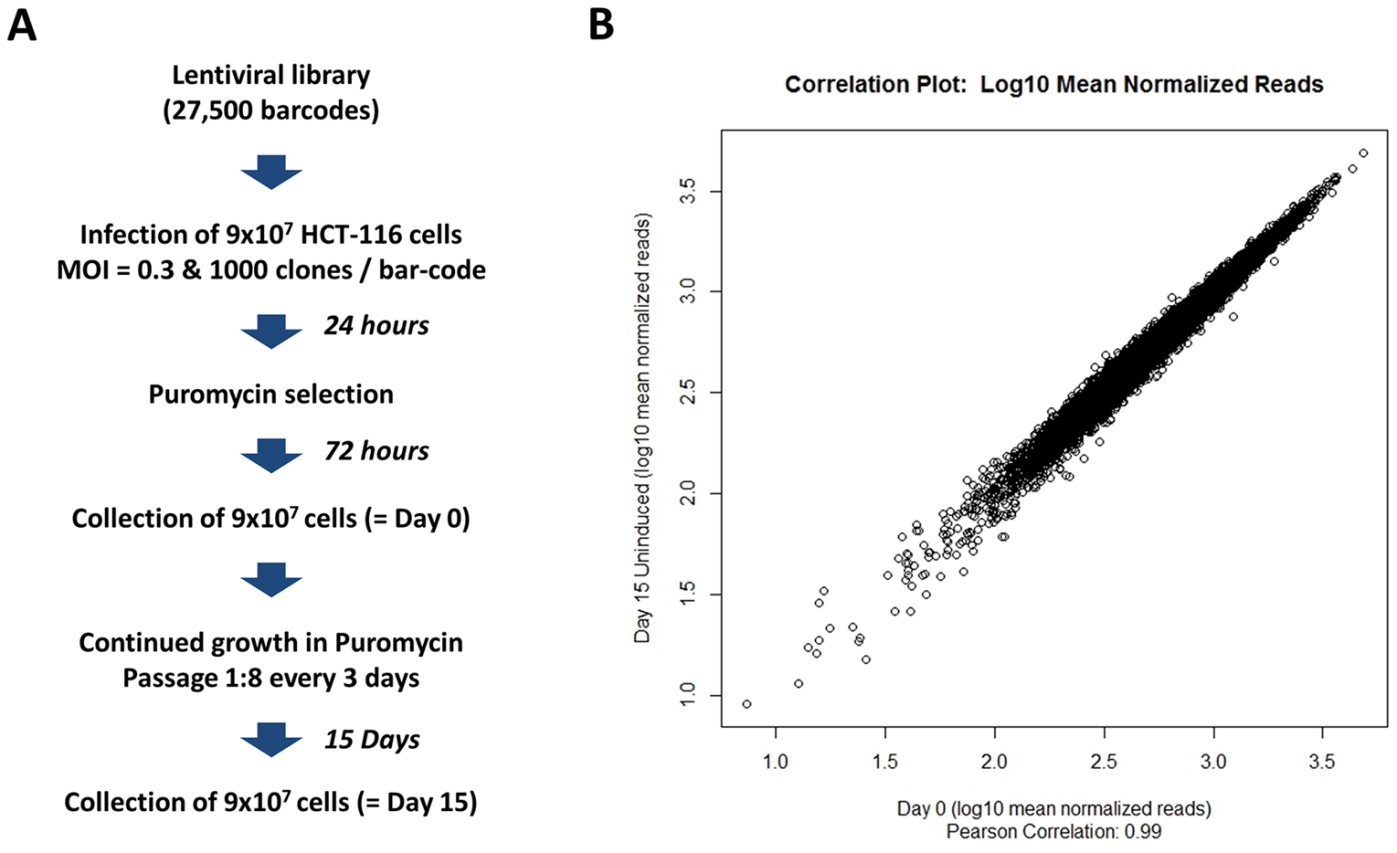
Evidence that encoded shRNA are not induced in the absence of Doxycycline *in vitro.* (A) A lentiviral library containing 27,500 unique barcodes was used to transduce HCT-116 cells at a MOI of 0.3. After 72 hours, of puromycin selection, cells were continuously passaged for 15 days in the absence of Doxycycline. Day 0 and Day 15 cell aliquots were obtained and total DNA from both time points were subjected to the barcode high-throughput sequencing retrieval procedure. (B) Correlation plot for Day 15 measurement against Day 0. shRNA barcode reads for all samples from all time points were first normalized to 2×10^7^ reads. Values for triplicate samples were averaged. At Day 15, strong repression of shRNA expression in the absence of Doxycycline is evident in the correlations with the Day 0 reference. Plot of log10 mean normalized reads for Day 0 against Day 15 (Pearson correlation: R = 0.99).

The shRNA encoded in the lentiviruses are cloned downstream of the U6-TET promoter and are regulated by a Tet-repressor encoded in the lentivirus, to ensure the repression of shRNA hairpin expression in the absence of Doxycycline. Thus, for the purpose of this experiment, the content of the lentiviral vectors was purely used as a bar-coding system of the infected cells and was not predicted to interfere with cell growth. To ensure that the encoded shRNA were not affecting cell growth, we grew the cells in a medium containing Tet-system approved FBS, designed to minimize de-repression of the promoters in the absence of Doxycycline. However, to confirm experimentally that unforeseen shRNA induction did not affect the barcode distribution in the absence of Doxycycline, we also compared the distribution of the barcodes contained in the KE-U6-TET library following HCT-116 transduction at Day 0 and at Day 15 post infection. For this experiment, we transduced HCT-116 cells with the KE-U6-TET lentiviral library at an MOI of 0.3 and collected cells right after infection (Day 0) or after 15 days of *in vitro* growth in the absence of Doxycycline (Day 15) (Fig. 3A). We observed a very tight correlation (R = 0.99) between the number of reads obtained for each shRNA barcode at Day 0 and Day 15 (Fig. 3B). We then evaluated if the two shRNA distributions at Day 0 (Fig. S1A), and Day 15 (Fig. S1B) were significantly different from each other using a non-parametric Kruskal-Wallis Rank Sum Test and obtained a P value of 0.972, indicating no statistical difference between the rank order of the different shRNA in the shRNA population at Day 0 and at Day 15 (Fig. S1D). Likewise there was no change in the overall distribution of shRNA between the two time-points, as established by a t-test p-value of 0.99 (Fig. S1D). These results indicate that in the absence of Doxycycline, the U6-TET-driven shRNA hairpins were not affecting the growth and distributions of barcodes in the transduced HCT-116 cell population *in vitro*, and were therefore unlikely to affect their distribution in the xenografts, provided that the hairpins remained uninduced *in vivo*.

**Figure 4:**
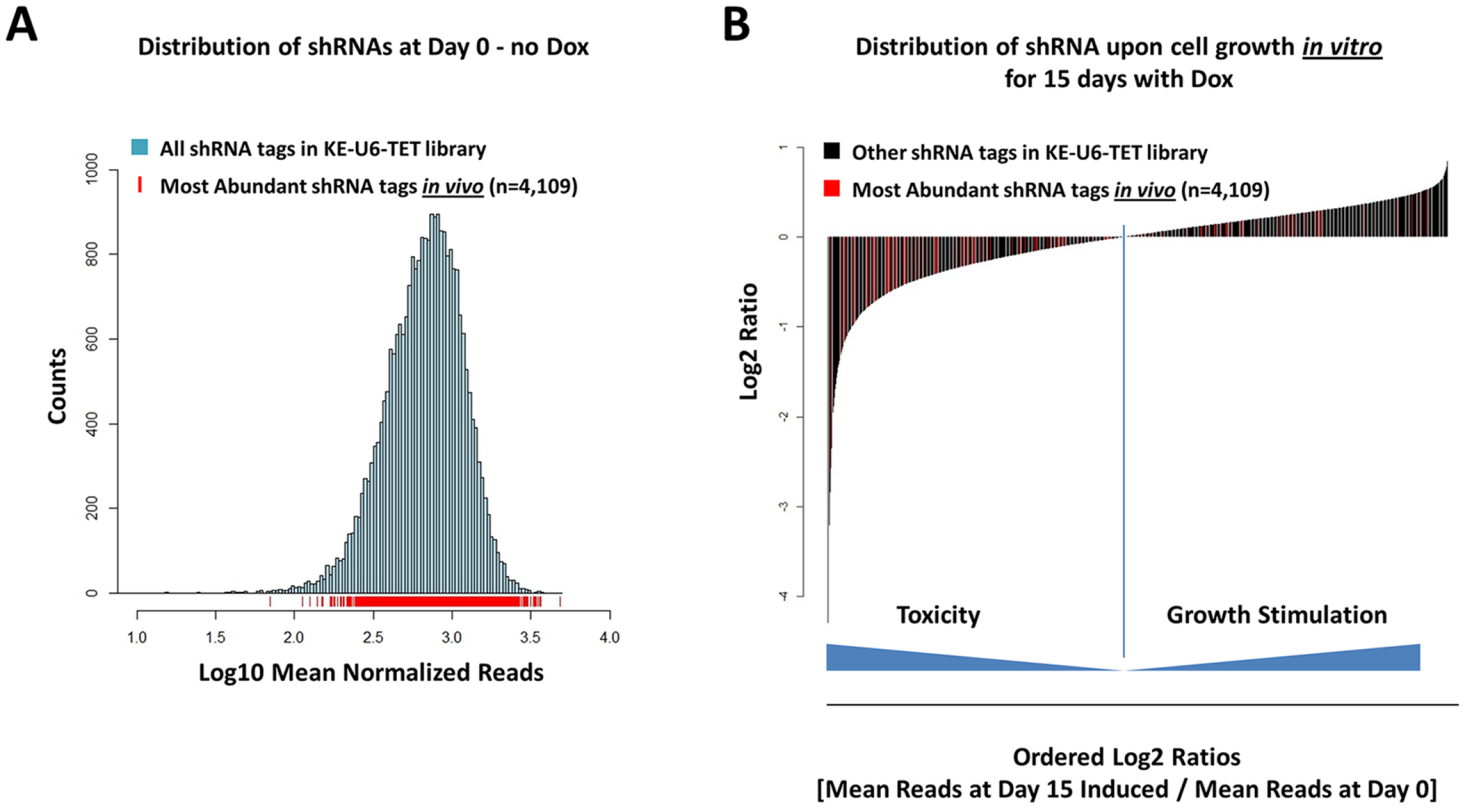
Evidence that encoded shRNA did not influence HCT-116 clonal cell growth *in vivo*. (A) Distribution of the 4,109 unique barcodes retrieved most abundantly from the three xenograft tumors (marked with red vertical lines) across the entire shRNA population distribution in the shRNA library. (B) Detection of a large fraction of the most abundant barcodes from the three xenograft tumors (marked in red) in the shRNA population most toxic to HCT-116 upon Doxycycline induction *in vitro* for 15 days (measured by a negative Log2 ratio of mean read counts for individual shRNA at Day 15 following Doxycycline to mean read counts at Day 0).

To model what would happen if the hairpins were de-repressed in a cancer cell population, we also compared the shRNA barcode distribution in transduced HCT-116 cells at Day 0 and at Day 15 following *in vitro* growth in the presence of Doxycycline (Day 15 - Dox - Fig. S1C). Under these conditions, while the overall shRNA population distribution was not significantly affected (p-value of a t-test between the two populations was 0.99), the rank-order of the different shRNA barcodes was profoundly affected (p-value of a non-parametric Kruskal-Wallis rank-order test was 1.75×10^−8^ - Fig. S1D). We then analyzed the distribution of the barcodes most abundantly identified in the three *in vivo* xenograft replicates (identified as part of the top 6% of clones contributing to 95% of the retrieved tags - see Fig. 2) to dispel two possible but trivial explanations for the clonal dominance we observed: (i) that of an initial barcode representation bias and (ii) the possible effect of leaky hairpins that would have benefited some clones over others *in vivo*. First, we did not observe a barcode distribution bias that would have favored the clones that were retrieved *in vivo*. The 4,109 most abundant shRNA barcodes retrieved in the three *in vivo* replicates were distributed across the entire shRNA population distribution and cannot account for the clonal dominance effect we observe (Fig. 4A). Second, if the leaky expression of shRNA hairpins *in vivo* was a factor in clonal dominance, we would expect that the clones that were selected *in vivo* would encode shRNA hairpins that favor HCT-116 growth, or at least, are not toxic to HCT-116. This was not the case: a large number of the most abundant barcodes that were retrieved *in vivo* (colored in red in Figure 4B) actually encode shRNA hairpins that are toxic to HCT-116 *in vitro*, as they actually cluster on the left-hand side of the shRNA distribution curve, indicating a growth inhibitory effect, after *in vitro* induction by Doxycycline for 15 days (Fig. 4B), strongly suggesting that these shRNA hairpins were not de-repressed during the growth of the engrafted tumor. Had these toxic shRNA hairpins been leaky or de-repressed, they would have hindered the growth of the clones that encoded them and their barcode would likely not have been amongst the most abundant shRNA barcodes retrieved *in vivo*.

### Clonal Dominance is Observed in Other Xenograft Models and in Another Mouse Host

To further validate our observations with additional cell lines and with a different host strain, we transduced 3×10^6^ HCT-116, MDA-MB-468 and A2780^cis^ cells in duplicate at an MOI of 0.18, 0.17 and 0.15, respectively, with a lentiviral library containing millions of unique barcodes (slightly different MOIs resulted from cell line to cell line variation in infectivity using the same lentiviral titer). These infections resulted in the transduction of 540,000 HCT-116 cells, 510,000 MDA-MB-468 cells and 450,000 A2780^cis^ cells that were incubated overnight prior to subcutaneous injection in Nude mice in a 1∶1 PBS:Matrigel solution. In the barcode library, 2% of the barcodes were associated with 27 neutral shRNAs directed at the Renilla *Luciferase* gene, which were previously tested in other cell lines *in vitro* and *in vivo* and were found to be non-toxic triggers (data not shown). Thus, initially, Nude mice injected with HCT-116, MDA-MB-468 and A2780cis respectively contained an estimated 10,800, 10,200 and 9,000 cells carrying unique barcodes associated with neutral shRNAs. Using applicable Poisson distribution calculations, fewer than 10% of these infected cells were predicted to carry more than one lentiviral insertion (see Methods). Xenograft tumors were allowed to grow for 14 days for HCT-116 and A2780^cis^ cells or for 24 days for the slower-growing MDA-MB-468. Xenografts were then resected, genomic DNA extracted and barcodes identified and counted following high-throughput sequencing. The barcode recovery rate for the duplicate sample for HCT-116 and A2780^cis^ were similar, while less consistent for the two MDA-MD-468 samples (Table 4). Subsequent graphs show the clonal distribution data for the first of each duplicate xenograft tumor, but the full data set of clones and barcode counts is available (Table S2).

**Table 4:** Barcode recovery rate in Athymic Nude xenograft experiments.

Cell Line	Replicate	Recovery rate (%)
HCT-116	1	*82*
	2	*72*
MDA-MB-468	1	*43*
	2	*19*
A2780^cis^	1	*62*
	2	*49*

**Figure 5:**
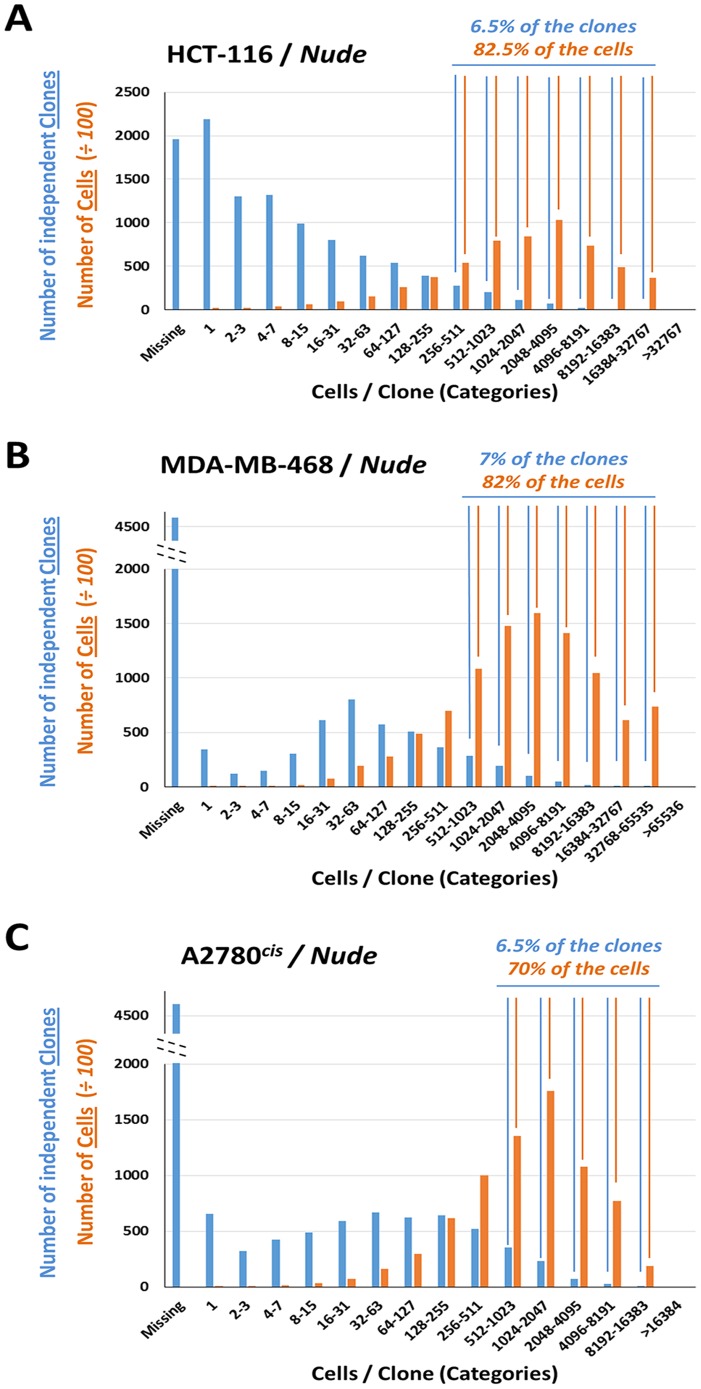
Distributions of HCT-116, MDA-MB-468 and A2780^cis^ barcoded cells upon *in vivo* growth in Nude mice. For each graph, the number of independent barcoded clones (blue bars) and the total aggregate number of barcoded cells (orange bars) are plotted for each clone size category across the X axis. (A) Distribution of barcoded HCT-116 cells: 82.5% of the barcoded cells were derived from 6.5% of the initially tagged and injected clones. (B) Distribution of barcoded MDA-MB-468 cells: 82% of the barcoded cells were derived from 7% of the initially tagged and injected clones. (C) Distribution of barcoded A2780^cis^ cells: 70% of the barcoded cells were derived from 6.5% of the initially tagged and injected clones. Scale for number of cells was adjusted 100 fold to allow side by side comparisons of clone numbers and cell numbers.

For each of the cell lines, the normalized barcode reads data expressed either as clone numbers or aggregate total cell numbers were plotted alongside each other as distributions across different categories of increasing cells per clone content (Fig. 5). In each case, the data clearly demonstrates that barcodes that mark independent clones are not represented evenly inside tumors, indicating a significant selection *in vivo,* even in populations of barcodes with no selective advantage. In each case, a small percentage of the initially injected clones (Fig. 5A-C - blue bars) contributed the vast majority of the descendant cells after 14 or 24 days of *in vivo* growth (Fig. 5A-C - orange bars), confirming that the phenomenon we termed clonal dominance can be observed in multiple Xenograft models (HCT-116, MDA-MB-468 and A2780^cis^) and in two different host strains (NSG and Athymic Nude mice).

## Discussion

### Factors Contributing to in vivo Clonal Dominance in Xenografts

We have defined clonal dominance as the result of remarkable heterogeneity in cell growth and tumor contribution from the individual constituent sub-clones within a population of cells injected subcutaneously; an intriguing result, given that HCT-116 xenografts are derived from a unique line thought to be clonal. Among the possible explanations for this phenomenon, we have discarded the possibilities of a barcode representation bias and the *in vivo* de-repression of the shRNAs encoded in the barcoded lentiviruses that could have theoretically led to the selection of particular clones (Fig. 4). Clonal dominance observed in HCT-116 cells implanted *in vivo* is also unlikely to be due to the presence of a small number of cancer initiating cells in the whole HCT-116 cell population, as this cell line has been shown to conform to a stochastic growth model, rather than a hierarchical stem-cell growth model. FACS sorted HCT-116 cells positive for known epithelial cancer stem-cell markers did not form tumors at a higher frequency than HCT-116 cells that did not carry these markers suggesting that the highly undifferentiated and aggressive HCT-116 cell line mostly consists of tumor-initiating cells [16], [17]. Clonal dominance could also theoretically result from stochastic lentiviral insertions in the HCT-116 genome, which may contribute to the selection of a small subset of clones *in vivo* upon disruption of haplo-insufficient tumor-suppressor genes, or activation of oncogenes. However, given the low number of inserts (one per cell on average and an N ∼ 10,000) and the fact that clonal dominance was not observed *in vitro*, we do not think random lentiviral insertions provide a satisfactory explanation for this phenomenon.

We have observed clonal dominance in several cancer xenograft models (HCT-116, MDA-MB-468 and A2780^cis^) and in two different mouse strains (NSG and Athymic Nude mice) and believe clonal dominance is most likely to stem from the interplay between two stochastic contributing factors. First,, even within a supposedly homogeneous cancer cell line population, cancer cells appear to possess different intrinsic replicative potentials with some clones nearer to the end of their potential than others: this is clearly evident in the *in vitro* data set, where clones with various replication potentials or phenotypic states clearly co-exist (see distribution of clones after 8 days of *in vitro* growth across multiple clone size categories in Fig. 2A - blue bars). The existence of these distinct phenotypic states within cell lines and the stochastic transition between two states has been elegantly modeled previously [18]. Second, there is an important stochastic positive selection *in vivo* for cells that are located in a favorable micro-environment (for example, in close proximity to blood vessels) and these clones appear to benefit disproportionately from their favorable initial spatial distribution (reviewed in [19]). Thus, a selection process within a genetically homogeneous clone appears to be primarily driven *in vivo* by randomness rather than genetics, a notion supported by a recent study studying serially transplanted xenografts showing that cancer cells from a particular initial genetic clone follow different growth patterns *in vivo* over time and oscillate between different behaviors, in an apparently stochastic fashion, such as stasis followed by growth, growth followed by stasis, continuous growth or growth arrest [20].

### Clonal Dominance and Tumor Initiating Potential Measurement

To determine the frequency of tumor initiating cells in a cancer cell population, the standard approach has been to use a limiting dilution technique that entails injecting lower and lower numbers of cells until the minimum number of cells required for tumor formation in mice is reached. For example, if you have to inject at least 100 cells to obtain a tumor, then the engrafting rate is assumed to be approximately 1 in 100 cells, or 1%. While the significance of these results regarding the existence of so-called “cancer stem cells” has been hotly debated [3], [21], [22], there clearly appears to be sub-populations of cells within tumors that are more adept at tumor engraftment in some mouse models than others [4].

However, this limiting dilution approach to determine the percent of cell engrafting is potentially problematic for two reasons. First, for some cell lines, highly diluted cells likely behave differently from less diluted cells [17]. As a result, the engrafting rate may not be linear with cell concentration as some cancer cells could engraft more easily with the support of more surrounding cells. Second, the approach assumes implanted cells only behave in a binary manner: either they implant and form a tumor or they don’t. In fact, our data and others [20] demonstrate that tumor engraftment is not a binary variable, but a continuous variable: some cells do not grow, but most contribute in varying degrees to the overall tumor mass. Measuring tumor engraftment *in vivo* could be improved with the use of complex clonal lentiviral libraries containing millions of unique barcoded lentiviral particles to insure that each lentivirally-transduced cancer cell carries a unique signature traceable by high-throughput sequencing. A population of cancer cells infected with such a clonal library and implanted *in vivo*, would contain millions of individually-traceable clones and would enable the accurate assessment of the contribution of each clone to the resulting tumor. In addition, different sub-populations of cancer cells FACS-sorted from patient tumors for particular markers (such as putative stem cell markers) could be tagged with different barcode sets and then re-implanted together into immune-deficient mice. With this approach, diverse cancer cell sub-population could be tracked simultaneously *in vivo* for their ability to compete with each other, undiluted, which would represent a much more physiological setting of cancer cell growth, given that various sub-populations of cells co-exist *in vivo*, not to mention that it would prove far easier than serially diluting individual sub-populations of cancer cells.

### Clonal Dominance and in vivo shRNA Screens

Another issue addressed by our data concerns the feasibility of running large-scale pooled shRNA dropout screens to identify genes required for viability *in vivo* using xenograft or allograft models. Our results indicate that it might be possible to perform an *in vivo* viability screen using a very small pooled shRNA library, provided that the cell are transduced in such a way that each shRNA hairpin is represented at least a 100 times into the highly proliferating fraction of each tumor. Designing such a library would require a prior knowledge of the percentage of injected cells that proliferate *in vivo* for a given cell line in a given host strain. In the case of HCT-116 cells tested in NSG mice, if we (i) set the “in vivo survival rate” at 6% (by including all the clones that contributed to 95% of the resulting barcoded cell within the tumor mass as shown in Figure 2B), (ii) inject 3×10^6^ cells per mice, (iii) use an MOI of 0.2 for the lentiviral library transduction to minimize the number of multiple transduction events at less than 10% of transduced cells, and (iv) want to have at least 100 “tumor contributing cells” per shRNA, we could probably screen 360 shRNA triggers (3×10^6^ * 0.2 * 0.06/100 = 360) in a pooled library setting. By using 8 shRNA triggers per gene, a number that should be considered a minimum, given the well-known off-target effects of individual shRNA triggers [23], [24], an *in vivo* screen could be run with a library designed to assess about 45 genes, a rather small number. Even with such small libraries, however, we predict that high clonal growth heterogeneity within the subset of “tumor contributing cells” would still make it very difficult to separate the signal from the noise without high numbers of replicates. If the of cells/shRNA ratio was to be decreased in order to accommodate increased number of shRNA triggers for a larger-scale pooled shRNA library targeting several hundred genes, the number of replicates needed to separate the signal from the noise would become prohibitive.

To illustrate the confounding variability introduced by clonal dominance *in vivo*, we randomly sampled 100 detected barcodes in the resulting *in vitro* and *in vivo* cell populations at the end-point of the HCT-116 experiment, thus simulating the fate of 100 cells independently transduced with neutral shRNAs. For 100 independent iterations of this sampling process, this simulation produces very reproducible aggregate cell counts in the three *in vitro* replicates (standard deviation ∼16–27%) (Fig. S2A). However, this random sampling process yielded highly variable counts in the three *in vivo* replicates (standard deviations >100%) (Fig. S2B). Thus, we conclude that high-throughput pooled shRNA drop-out screens are likely to yield highly variable shRNA barcode counts *in vivo*. High variability was indeed documented in one prior study using a syngeneic *Eµ-myc* lymphoma model [5]. However, in a study using an orthotopic xenograft model of MCF10.DCIS breast cancer cells, the use of relatively small libraries (235 and 516 shRNAs, respectively) and relatively high numbers of replicate (5 and 12), reported a more manageable variability [6], as we would predict.

We believe an approach in which each infected cell can be traced with a unique barcode has the potential to circumvent the confounding effect of clonal dominance while allowing for the use of large shRNA libraries *in vivo*. By using a reasonably small set of barcode tags on the vector and the shRNA oligonucleotide inserts, it is possible to create a “double barcode” system with sufficient complexity to uniquely identify the shRNA triggers and at the same time uniquely label each cell upon transduction. This approach provides a way of tracing the fate of individual clones in a tumor *in vivo*. Furthermore, since each shRNA sequence is represented by independently barcoded clones, the “clonal dominance” effect can be circumscribed: the output of a clonal tagging and tracking experiment are clonal distributions of numerous individual clones for the same shRNA sequence, rather than aggregate counts of all the cells for each shRNA, which are highly subject to the distorting effect of clonal dominance *in vivo*. In other words, the effect of shRNA expression would be independently measured in each and all transduced clones for each shRNA. Using such a design, 3×10^6^ HCT-116 cells infected at an MOI of 0.2 and injected subcutaneously could be screened *in vivo* with a library of 6,000 shRNA, if each shRNA sequence is to be represented by 100 independent sub-clones (3×10^6^ * 0.2/100 = 6,000). Using 8 shRNA per gene, a library could be designed to assess the function of 750 genes. Because this approach is scalable, in theory, libraries of up to several thousand genes could be envisioned.

In conclusion, we have shown that barcoding individual cancer cells within a population prior to subcutaneous injection allows for the tracking of the fate of individual clones *in vivo* using high-throughput DNA sequencing technology. Our results demonstrate that a minority of the barcoded clones contribute disproportionately to the descendant cells within the tumor mass, a stochastic effect we have termed “clonal dominance”. This observation calls into question the feasibility of large-scale shRNA screens *in vivo* using traditional pooled shRNA library designs and highlights the need to closely assess the heterogeneous growth characteristics of any putative tumor model when planning such functional screens *in vivo*. We propose using clonal lentiviral libraries to individually track all the barcoded cells within a cancer cell population as a way to perfect *in vivo* engraftment measurement and shRNA library screens *in vivo*.

## Supporting Information

Figure S1 Comparison of the shRNA distributions in the presence or absence of Doxycycline induction. Histograms of log10 mean normalized shRNA barcode reads for (A) reference measurement at day 0, (B) measurement after 15 days of in vitro growth without Dox, (C) measurement after 15 days of in vitro growth in the presence of Dox. (D) A Kruskal-Wallis rank sum test and a T-test were performed comparing the ranked distributions and the means normalized reads from Day 15 with or without Doxycycline induction and the Day 0 reference.
(TIF)

Figure S2 Lack of clonal growth reproducibility *in vivo.* For each in vitro and in vivo replicate, the sum of cell counts for 100 detected independent barcoded clones selected at random was averaged for 100 independent sampling iterations. (A) In vitro, the obtained average aggregated cell counts in the three independent replicates presented a 16–27% Standard Deviation. (B) In vivo, the obtained average aggregated cell count in the three independent replicates presented a Standard Deviation >100%, indicating very high variability in the in vivo data set.
(TIF)

Table S1 Normalized barcode counts for HCT-116 cells grown in vitro or as xenografts in NSG mice. The normalized number of barcodes for each shRNA lentivirus in the KE-U6-TET library retrieved after high throughput sequencing of infected HCT-116 cells upon *in vitro* growth or *in vivo* growth in NSG mice.
(XLSX)

Table S2 Normalized barcode counts for cancer cells grown as xenografts in Nude mice. The normalized number of barcodes for each neutral *Luciferase* shRNA lentivirus retrieved after high throughput sequencing of infected MDA-MB-468, HCT-116 or A2780^cis^ cells upon *in vivo* growth in Nude mice.
(XLSX)
