# Supplementary figures and images for: Measurement of Cancer Cell Growth Heterogeneity through Lentiviral Barcoding Identifies Clonal Dominance as a Characteristic of In Vivo Tumor Engraftment
Source: PLoS One. 2013 Jun 26;8(6):e67316. doi: 10.1371/journal.pone.0067316 (PMC3693957; doi:10.1371/journal.pone.0067316)

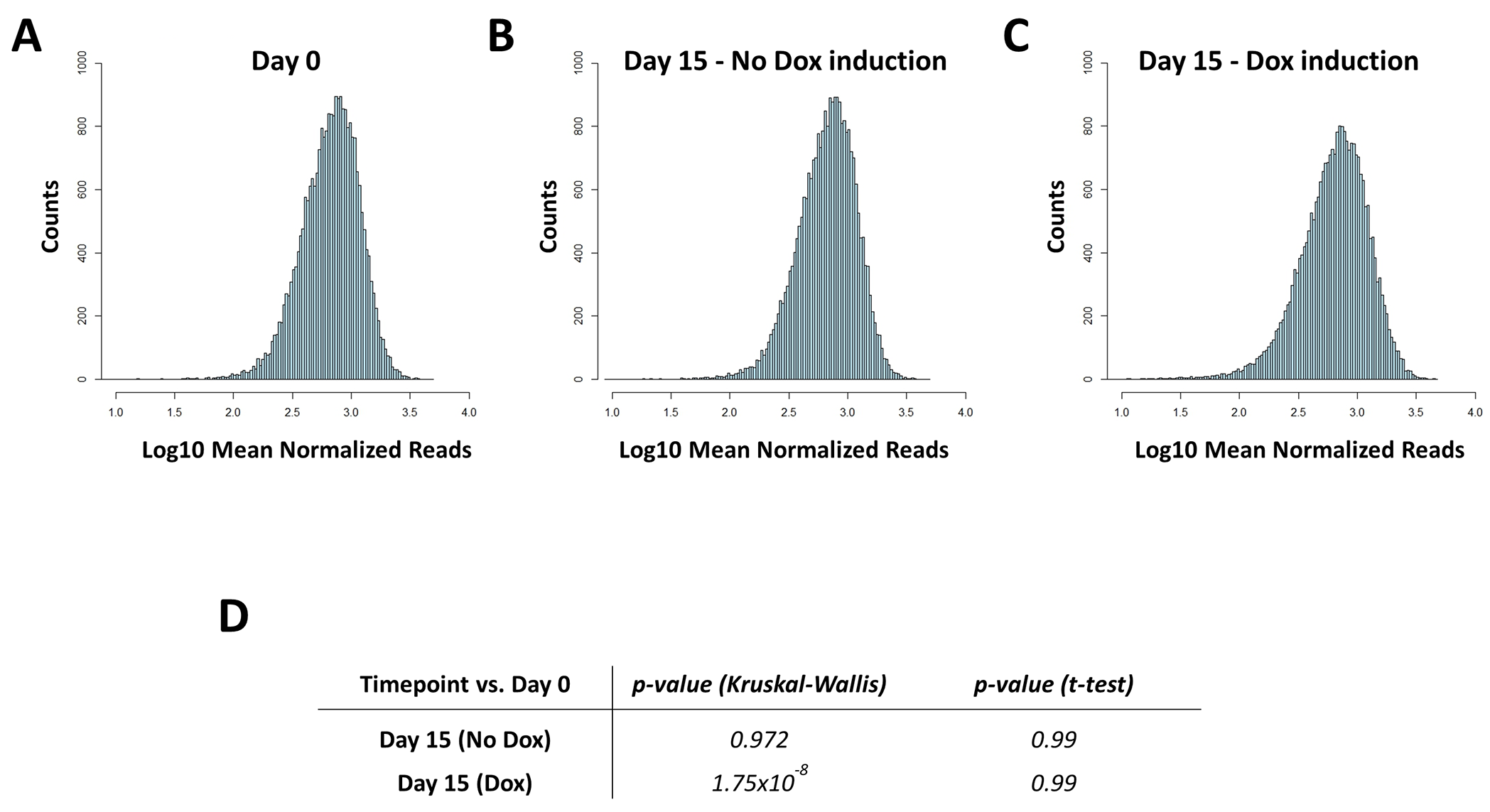

Supplement: Figure S1 — Comparison of the shRNA distributions in the presence or absence of Doxycycline induction. Histograms of log10 mean normalized shRNA barcode reads for (A) reference measurement at day 0, (B) measurement after 15 days of in vitro growth without Dox, (C) measurement after 15 days of in vitro growth in the presence of Dox. (D) A Kruskal-Wallis rank sum test and a T-test were performed comparing the ranked distributions and the means normalized reads from Day 15 with or without Doxycycline induction and the Day 0 reference. (TIF) [file pone.0067316.s001.tif]

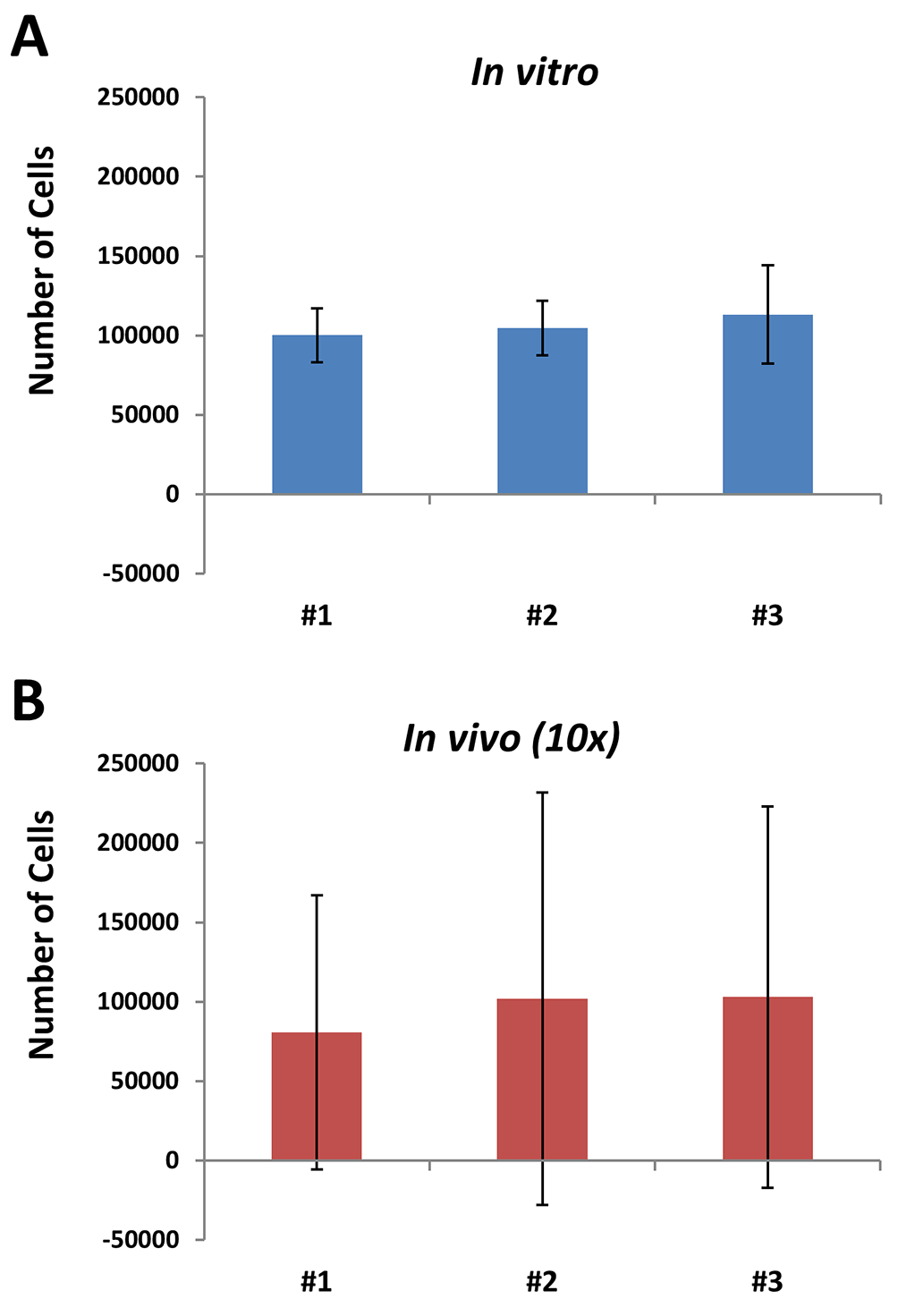

Supplement: Figure S2 — Lack of clonal growth reproducibility in vivo. For each in vitro and in vivo replicate, the sum of cell counts for 100 detected independent barcoded clones selected at random was averaged for 100 independent sampling iterations. (A) In vitro, the obtained average aggregated cell counts in the three independent replicates presented a 16–27% Standard Deviation. (B) In vivo, the obtained average aggregated cell count in the three independent replicates presented a Standard Deviation >100%, indicating very high variability in the in vivo data set. (TIF) [file pone.0067316.s002.tif]
